# Comparison of systemic treatments for previously treated patients with unresectable colorectal liver metastases: a systematic review and network meta-analysis

**DOI:** 10.3389/fonc.2024.1293598

**Published:** 2024-07-10

**Authors:** Yunlin Jiang, Mingye Zhao, Wenxi Tang, Xueping Zheng

**Affiliations:** ^1^ Nanjing Hospital of Chinese Medicine Affiliated to Nanjing University of Chinese Medicine, Nanjing, China; ^2^ Graduate School of Nanjing University of Chinese Medicine, Nanjing, China; ^3^ Department of Pharmacoeconomics, School of International Pharmaceutical Business, China Pharmaceutical University, Nanjing, Jiangsu, China

**Keywords:** metastatic colorectal cancer, unresectable liver metastases, network meta-analysis, treatment selection, overall survival

## Abstract

**Background:**

There is limited evidence of comparative results among different treatments for patients with unresectable colorectal liver metastases (CRLM) who have failed at least one line of previous systemic therapy. We aimed to compare the efficacy of systemic treatments among these patients through this investigation.

**Methods:**

We collected randomized controlled trials (RCTs) reported in English up until July 2023, from databases including PubMed, Embase, Cochrane Library, ClinicalTrials.gov, and prominent conference databases, for this Bayesian network meta-analysis. Phase II or III trials that evaluated at least two therapeutic regimens were included. Primary outcome was overall survival (OS), secondary outcome was progression-free survival (PFS). Hazards ratios (HRs) with 95% confidence intervals (CIs) were used as effect size. Subgroup analysis was performed based on metastatic sites. The current systematic review protocol was registered on PROSPERO (CRD42023420498).

**Results:**

30 RCTs were included, with a total of 13,511 patients. Compared to chemotherapy, multi-targeted therapy (HR 0.57, 95% CI 0.37–0.87) and targeted therapy plus chemotherapy (HR 0.78, 95% CI 0.67–0.91) show significant advantages. Targeted therapy (HR 0.92, 95% CI 0.54–1.57) and local treatment plus chemotherapy (HR 1.03, 95% CI 0.85–1.23) had comparable performance. For patients with liver metastases, TAS-102 plus bevacizumab, aflibercept plus fluorouracil-based combination chemotherapy (CTFU), and bevacizumab plus capecitabine-based combination chemotherapy (CTCA) showed the best outcomes in terms of OS. Bevacizumab plus intensified CTFU, bevacizumab plus CTCA, and HAI followed by single-agent chemotherapy (SingleCT) performed the best regarding PFS. For patients with liver-limited metastases, aflibercept plus CTFU is the optimal choice in OS. For PFS, the best options were HAI followed by SingleCT, aflibercept plus CTFU, and panitumumab plus CTFU. For patients with multiple-site metastases, the best treatments were TAS-102 plus bevacizumab, bevacizumab plus CTCA, bevacizumab plus CTFU, and aflibercept plus CTFU.

**Conclusion:**

Multi-targeted therapy and targeted therapy plus chemotherapy are the best treatment mechanisms. TAS-102 plus bevacizumab is superior in OS, the combination of anti-VEGF drugs like bevacizumab and aflibercept with standard chemotherapy is the preferred option for CRLM patients.

## 1 Introduction

Colorectal cancer (CRC) is responsible for around 10% of all cancers diagnosed globally every year, making it the second most prevalent cancer in women and the third most common in men (1). More than half of all CRC patients develop metastases, with the liver being the most common site for distant metastasis (2, 3). Factors associated with colorectal liver metastases (CRLM) may include: the connection of the portal vein system between the colorectal and liver, which provides a rich blood supply; as well as the location and histological type of the primary tumor. Liver metastases are developed in approximately 50% of patients diagnosed with CRC, and synchronous liver metastases are present in 10–15% of patients (4, 5).

For patients with CRLM, curative resection is considered a standard approach (6). The application of this method is limited to only 20–30% of cases because of factors like tumor location, size, patients’ comorbidities, or unresectable disease. As a result, the 5-year survival rate decreases significantly to around 30% (7, 8). The efficacy of radiation therapy for CRLM patients is subject to debate, mainly due to the liver’s low tolerance for radiation doses compared to the higher doses needed to eradicate tumor cells. Nevertheless, percutaneous radiation treatments such as proton therapy and CyberKnife can be highly effective for patients with a few small, strategically located liver metastases. These therapies provide highly precise targeting, thereby minimizing damage to the surrounding healthy tissues. Conventional radiotherapy techniques are suitable for treating CRLM patients with normal liver function (9, 10). To enhance control over local lesions, suitable ablation treatments could be chosen based on location, treatment goals, and complications when surgical removal of liver metastases is not feasible (11). Systemic chemotherapy is typically given to patients with CRLM who are ineligible for resection (12–14). In recent decades, research has shown that patients with CRLM who received systematic chemotherapy experienced a significant improvement in their overall survival (OS) (15, 16). Regional therapies, including hepatic arterial infusion (HAI), conventional transarterial chemoembolization (cTACE), and transarterial radioembolization (TARE), serve as valuable alternatives for patients who exhibit limited response to initial chemotherapy. Particularly in cases of liver-only metastasis, it is essential to assess the number, size, and location of the tumors to effectively tailor the treatment approach. Integrating systemic chemotherapy with regional and percutaneous therapies often leads to superior outcomes. This holistic approach maximizes the benefits of each treatment modality, enhancing the precision and efficacy of the therapy, and ultimately improves patient prognosis (17). During the process of metastasis, tumors enhance their energy supply by promoting angiogenesis, which is why anti-angiogenesis therapy is crucial for the treatment of CRLM. In particular, bevacizumab and cetuximab have been created as molecularly targeted medications (18). According to Saltz et al., the efficacy of incorporating bevacizumab into XELOX or FOLFOX-4 was evaluated in 1401 CRLM patients (19). In the bevacizumab group, the median duration of progression-free survival (PFS) was 9.4 months, which was significantly longer than that of the placebo group (P = 0.0023). Furthermore, the addition of cetuximab to FOLFOX-4, in comparison to using FOLFOX-4 alone, resulted in a significant improvement in overall response. For patients with initially unresectable CRLM, the combination of targeted drugs and chemotherapy can also result in a higher rate of remission and enhanced resectability (20). As more advancements are made in understanding immune checkpoint in various cancer types, particularly in DNA mismatch repair defects (dMMR)/high microsatellite instability (MSI-H) CRC, immunotherapy has emerged as an appealing treatment option alongside targeted therapy (5).

Currently, there are multiple options available for previously treated patients with unresectable CRLM. Meanwhile, there is a lack of relative outcomes among these treatments. Therefore, we conducted this network meta-analysis (NMA) of randomized controlled trials (RCTs) to systematically compare the efficacy of all current treatment regimens on patients with unresectable CRLM who have failed at least one previous line of systemic therapy, and to offer healthcare clinicians, patients, and relevant guidelines with references in clinical medication and disease management.

## 2 Methods

We conducted our study in accordance with the guidelines outlined in the extension statement of the Preferred Reporting Items for Systematic Reviews and Meta-Analyses (PRISMA) (21). See **Supplementary File 1**. This systematic review protocol was registered on PROSPERO (CRD42023420498).

### 2.1 Data sources and search strategy

The search strategy is provided in **Supplementary File 2**. On July 31, 2023, we conducted thorough search included PubMed, EMBASE, Cochrane Library, and ClinicalTrials.gov to identify relevant RCTs and published studies. We did not impose any limitations on the publication date, and only considered studies published in English. Additionally, we incorporated abstracts from the European Society for Medical Oncology, American Society of Clinical Oncology starting from 2021.

### 2.2 Selection criteria

Initially, titles and abstracts of the included articles were screened by two researchers. The eligibility criteria, which were based on the PICOS framework, were as follows:

(1) Population: Adult patients diagnosed histologically or cytologically with confirmed unresectable CRLM. Meanwhile, patients should receive at least one form of systemic treatment previously. No restrictions were placed on individual-level characteristics. Due to limited reporting in some RCTs, we assumed that patients who had metastases in two or more sites including liver metastases. Considering that the proportion of liver metastases reaches over 90% in patients with metastases in two or more sites (22, 23).

(2) Interventions and comparisons: We evaluated various systematic interventions, including pharmaceutical, surgical, radiological, and multi-mechanism therapies.

(3) Outcomes: Trials that reported hazard ratios (HR) of either OS or PFS.

(4) Study design: Phase II or III studies that evaluated various contrasting treatments were taken into account.

We only considered trials that offered the most recent and informative data to prevent repetition. Moreover, trials that investigated treatments unrelated to any comparisons were disregarded. Additionally, trials that explored different dosages but with the same administrations were also eliminated.

### 2.3 Data extraction and quality assessment

Two independent researchers (YJ and MZ) were responsible for extracting the required data. Any discrepancies that arose were resolved through discussions that involved other researchers (YJ, MZ, WT, and XZ). The extracted information was the characteristics of eligible trials (publication year, registration information, etc.), characteristics of populations (age, sample size, countries, etc.), and characteristics of the program (interventions, outcomes of endpoints, etc.). The clinical outcomes extracted included OS and PFS. The individual patient data from studies that only presented Kaplan-Meier curves without HR or 95% confidence intervals (CIs), was obtained using the tool developed by Liu et al. (24).

Quality of the studies was assessed using the risk of bias (ROB) tool from the Cochrane Collaboration (25). The eligible studies were divided into three categories based on their risk level: high, low, or uncertain (26). The Egger regression test was used to assess the presence of publication bias, considering p-values less than 0.10 as indicating biased results (27).

### 2.4 Statistical analyses

In this meta-analysis, the primary outcome was OS, while PFS served as a secondary outcome. Network plots were used to compare and visually display the various treatment options. Pooled HRs with 95% CIs were calculated for OS, PFS. In order to examine the synthesized HRs, the fixed effects consistency model was chosen because most of the direct evidence came from a single trial (28). Using the R package Gemtc, the Bayesian network meta-analysis (NMA) was carried out. A total of 50,000 samples were used, divided into four sets of Markov chains. Each set included 10,000 burn-in samples. Non-informative uniform and normal prior distributions were utilized (29). In addition, we performed calculations to determine the probability ranking for all available treatments and presented it using the surface under the cumulative ranking (SUCRA). A higher SUCRA value indicated a higher ranking.

The I^2^ statistic was used to assess the heterogeneity among the studies, with a moderate level of heterogeneity indicated by a value above 50% (29). Both direct and indirect evidence were considered when assessing the inconsistency of models using the edge-splitting method (29). In order to ensure the reliability of this study, numerous pairwise meta-analyses were conducted for comparison. The convergence of Markov chains was verified by utilizing Gelman-Rubin diagnostic statistics and trace plots (30).

In order to assess the robustness and reliability of the findings, and to assess the influence of metastatic sites, we performed subgroup analyses. We categorized the population into two groups: patients with liver-limited metastases and those with multiple-site metastases.

## 3 Results

### 3.1 Characteristics of the included studies

**Figure 1:**
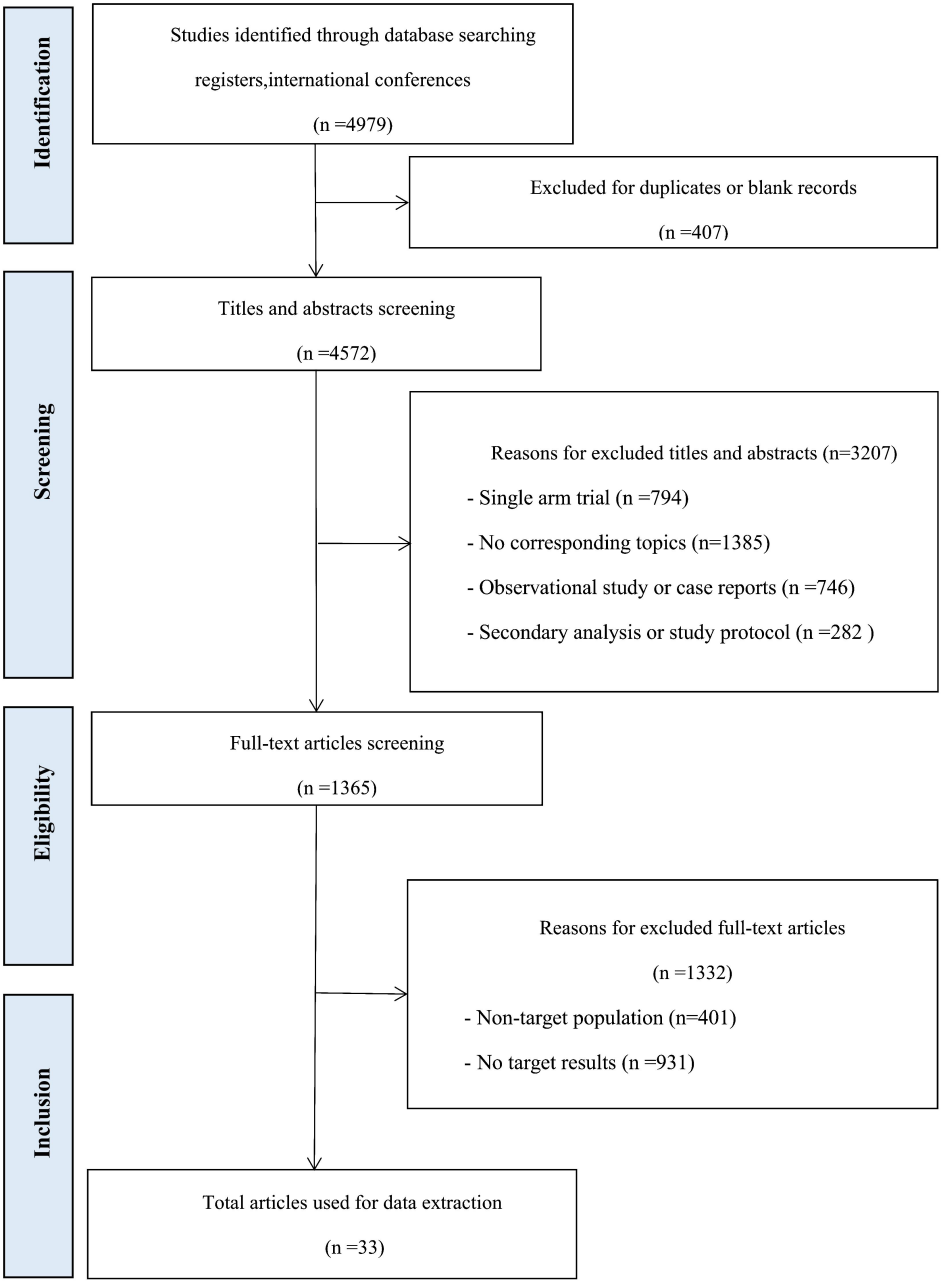
Study flow chat.

A total of 4979 records were obtained from the previously mentioned databases, out of which 1365 studies were determined suitable for full-text evaluation. Eventually, the analysis comprised of 30 RCTs, which were represented by 33 articles. The flow chart in **Figure 1** illustrates this process. The characteristics of the included studies can be found in **Table 1**. This research study included a total of 13,511 patients diagnosed with mCRC. In order to form a comprehensive comparison, we divided chemotherapy into single-agent chemotherapy (SingleCT), fluorouracil-based combination chemotherapy (CTFU, defined as mFOLFOX6, FOLFOX4, FOLFOX, FLOX, FOLFIRI, fluorouracil/irinotecan, fluorouracil/leucovorin, or fluorouracil/oxaliplatin), capecitabine-based combination chemotherapy (CTCA, defined as CAPEOX, XELOX, OXXEL, XELIRI or capecitabine/mitomycin), and intensified CTFU (ICTFU, defined as FOLFIRINOX or mFOLFOXIRI). We unified best supportive care (BSC) and placebo as the same. There were 23 treatments involved, comprising aflibercept plus CTFU, anlotinib, bevacizumab plus CTCA, bevacizumab plus CTFU, bevacizumab plus ICTFU, BSC, cetuximab, CTCA, CTFU, famitinib, fruquintinib, HAI followed by SingleCT, napabucasin, nintedanib, panitumumab, panitumumab plus CTFU, ramucirumab, regorafenib, simvastatin plus CTFU, TACE plus CTFU, TARE plus CTFU, TAS-102 (trifluridine/tipiracil), and TAS-102 plus bevacizumab. Six treatment mechanisms were covered, including multi-targeted therapy, targeted therapy plus chemotherapy, targeted therapy, local treatment plus chemotherapy, chemotherapy, and BSC. For multi-targeted therapy, targeted therapy, and local treatment, the definitions are as follows: Multi-targeted therapy combines various agents and approaches to target tumor growth from multiple angles, integrating systemic chemotherapy with specific molecular-targeted drugs and regional treatments. Targeted therapy employs drugs that specifically attack cancer cells based on unique molecular markers, such as angiogenesis inhibitors, thereby minimizing damage to normal cells. Local treatment directly targets the liver tumor, using methods such as surgical resection, radiofrequency ablation (RFA), or transarterial approaches like TACE and TARE to deliver treatments directly to the tumor site.

**Table 1:** Characteristics of the included studies.

Trail	Study arms		Number of patients		Age (Mean, range or SD)		Sex (Male, %)		ECOG (0/1,%)		Proportion of liver metastasis (%)		Outcome
	T	C	T	C	T	C	T	C	T	C	T	C	
FRESCO-2 (31)	Fruquintinib	Placebo/BSC	461	230	64 (56–70)	64 (56–69)	53	61	100	100	74	68	PFS, OS
Yasutosh,2023 (32)	Bevacizumab plus CTFU	TAS-102 plus bevacizumab	199	197	68 (32–82)	67 (25–84)	50	58	100	100	59	65	OS
ALTER0703 (33)	Anlotinib	Placebo/BSC	282	137	NA	NA	63	66	100	100	77	70	PFS, OS
Pfeiffer,2020 (34)	TAS-102 plus bevacizumab	TAS-102	46	47	64 (57–69)	67 (58–72)	52	64	NA	NA	61	85	PFS, OS
Filippo,2020 (35)	CTCA	CTFU	43	43	70 (63–75)	67 (61–73)	42	56	100	100	NA	NA	PFS, OS
LUME-Colon 1 (36)	Nintedanib	Placebo/BSC	386	382	62 (22–85)	62 (23–83)	61	57	99.8	100	72	70	PFS, OS
FRESCO (37)	Fruquintinib	Placebo/BSC	278	138	55 (23–75)	57 (24–74)	57	70	100	100	67	73	PFS, OS
AXEPT (38)	Bevacizumab plus CTCA	Bevacizumab plus CTFU	326	324	61 (52−67)	60 (51−68)	60	58	99	99	64	59	PFS, OS
Jonker,2018 (39)	Napabucasin	Placebo/BSC	138	144	64 (32–85)	64 (37–81)	66	65	100	100	73	71	PFS, OS
Xu,2017 (40)	Famitinib	Placebo/BSC	99	55	55 (24–70)	54 (32–71)	57	60	100	100	NA	NA	PFS
RAISE (41, 42)	Ramucirumab	Placebo/BSC	536	536	62 (21–83)	62 (33–87)	54	61	99	99	NA	NA	PFS, OS
Lim,2015 (43)	Simvastatin plus CTFU	CTFU	134	135	57 (32–79)	57 (35–82)	58	67	99	99	60	63	PFS, OS
Peeters,2010WT (44, 45)	Panitumumab plus CTFU	CTFU	303	294	60 (28–84)	61 (29–86)	62	65	95	93	68	64	PFS, OS
Peeters,2010 Mu (44, 45)	Panitumumab plus CTFU	CTFU	238	248	61 (29–83)	64 (29–86)	56	60	94	94	70	69	PFS, OS
Peeters,2014 (46)	Panitumumab plus CTFU	CTFU	303	294	NA	61 (55- 68)	61	65	96	93	68	69	PFS, OS
SUNLIGHT (47)	TAS-102 plus bevacizumab	TAS-102	246	246	62 (20–84)	64 (24–90)	50	55	100	100	NA	NA	PFS, OS
TERRA (48)	TAS-102	Placebo/BSC	271	135	58 (26–81)	56 (24–80)	63	62	100	100	NA	NA	OS
Rothenberg,2008 (49)	CTCA	CTFU	313	314	61 (26–81)	60 (26–83)	62	61	92	93	NA	NA	PFS
Kim,2016 (50)	Panitumumab	Placebo/BSC	189	188	62 (30–82)	60.0 (19–79)	57	58	91	89	NA	NA	PFS, OS
BEBYP (51)	Bevacizumab plus CTFU	CTFU	92	92	62 (38–75)	67 (38–75)	57	75	98	99	NA	NA	PFS
CONCUR (52)	Regorafenib	Placebo/BSC	136	68	58 (50–66)	56 (49–62)	63	49	100	100	NA	NA	PFS, OS
EPOCH (53)	TARE plus CTFU	CTFU	215	213	63	60	63	65	NA	NA	100	100	PFS, OS
Liu,2021 (17)	TACE plus CTFU	CTFU	85	83	56 (11)	58 (11)	67	72	NA	NA	100	100	PFS, OS
HEARTO (54)	HAI followed by oxaliplatin	CTCA	16	11	66 (45–82)	55 (40–82)	56	64	100	100	NA	NA	PFS, OS
SPIRITT (55)	Panitumumab plus CTFU	Bevacizumab plus CTFU	91	91	60 (27–84)	60 (25–80)	68	64	99	100	NA	NA	PFS, OS
ASPECCT (41, 56)	Panitumumab	Cetuximab	499	500	61 (54–67)	61 (53–68)	63	64	92	92	NA	NA	PFS, OS
VELOUR (57)	Aflibercept plus CTFU	CTFU	186	187	59 (32–81)	60 (27–86)	59	56	97	97	79	78	PFS, OS
ML18147 (58)	Bevacizumab plus CTFU	CTFU	409	411	63 (27–84)	63 (21–84)	65	63	95	95	NA	NA	OS
20020408 (59)	Panitumumab	Placebo/BSC	231	232	62 (27–82)	63 (27–83)	63	64	87	84	NA	NA	PFS
AIO KRK 0314 (60)	Panitumumab plus CTFU	CTFU	70	36	61 (43–81)	65 (44–77)	71	86	99	100	NA	NA	PFS, OS
TRIBE2 (15)	Bevacizumab plus ICTFU	Bevacizumab plus CTFU	339	340	60 (53–67)	61 (52–67)	53	61	100	100	NA	NA	PFS, OS

BSC, best supportive care; CTCA, capecitabine-based combination chemotherapy; CTFU, fluorouracil-based combination chemotherapy; ICTFU, intensified CTFUTAS-102, trifluridine/tipiracil; SingleCT, Single-agent chemotherapy. NA, not available.

### 3.2 Risk of bias

The assessment of ROB is presented in **Supplementary File 3**. Overall, ROB in all RCTs was generally low. However, multiple RCTs were open-label (15, 18, 32, 34, 35, 38, 44, 46, 49–51, 53–55, 58–61), thereby raising concerns about participant and personnel blinding, outcome assessment. The results of the Egger test indicated no publication bias in our network, the funnel plots are displayed in **Supplementary File 4**.

### 3.3 Efficacy outcomes

#### 3.3.1 Liver metastases

**Figure 2:**
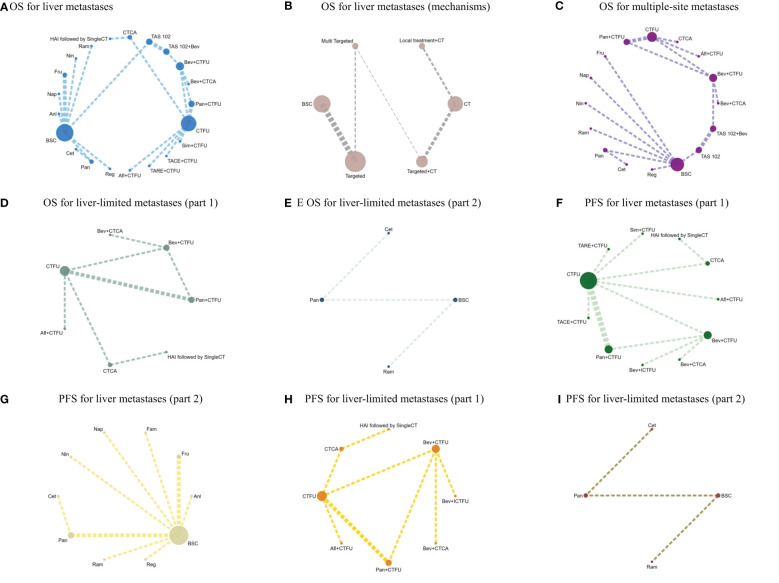
**(A)** OS for liver metastases; **(B)** OS for liver metastases (mechanisms); **(C)** OS for multiple-site metastases; **(D)** OS for liver-limited metastases (part 1); **(E)** OS for liver-limited metastases (part 2); **(F)** PFS for liver metastases (part 1); **(G)** PFS for liver metastases (part 2); **(H)** PFS for liver-limited metastases (part 1); **(I)** PFS for liver-limited metastases (part 2).

**Figure 3:**
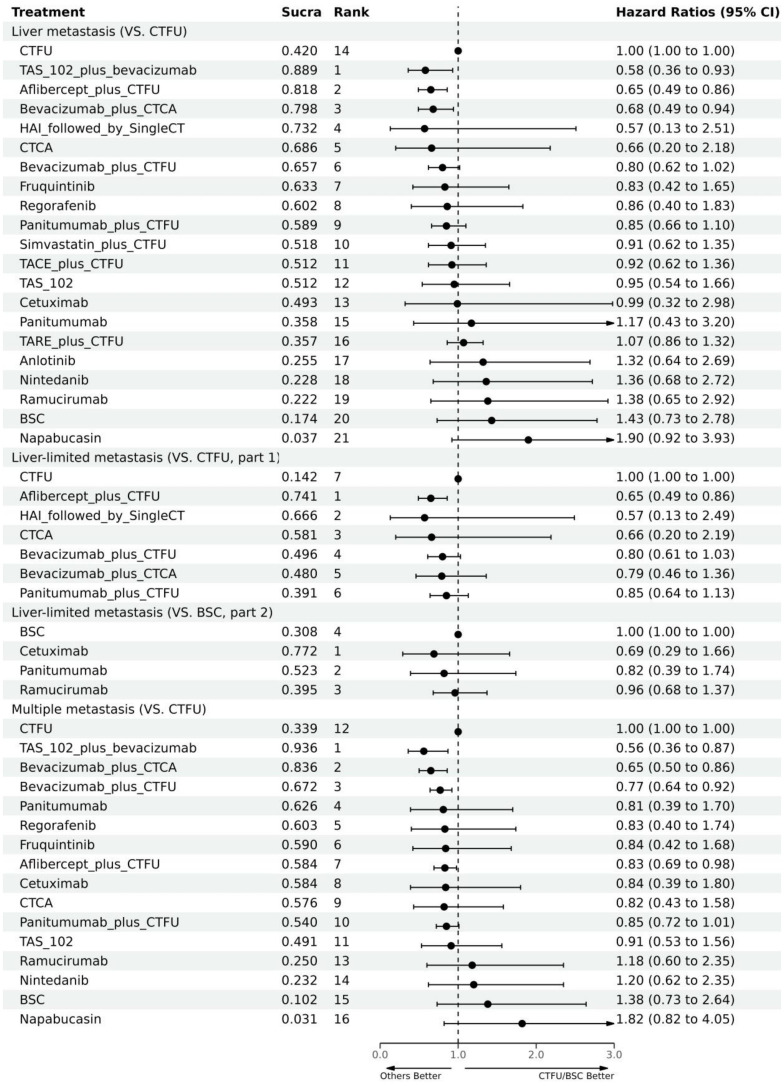
Forest plot for OS. CTFU, fluorouracil-based combination chemotherapy; CTCA, capecitabine-based combination chemotherapy; ICTFU, intensified chemotherapy containing four interventions; SingleCT, single-agent chemotherapy.

For OS, network plot is provided in **Figures 2A, B**. Among the 21 intervention options for patients with liver metastases, the top three ranked were TAS-102 plus bevacizumab (SUCRA 0.889), aflibercept plus CTFU (SUCRA 0.818), and bevacizumab plus CTCA (SUCRA 0.798). When compared to CTFU, treatments that had significant advantages were ranked from best to worst as follows: TAS-102 plus bevacizumab (HR 0.58, 95% CI 0.36–0.93), aflibercept plus CTFU (HR 0.65, 95% CI 0.49–0.86), and bevacizumab plus CTCA (HR 0.68, 95% CI 0.49–0.94). No significant difference in comparison to CTFU for the other options. Among the six mechanisms, the rankings from highest to lowest were as follows: multi-targeted therapy (SUCRA 0.985), targeted therapy plus chemotherapy (SUCRA 0.746), targeted therapy (SUCRA 0.503), local treatment plus chemotherapy (SUCRA 0.286), chemotherapy (SUCRA 0.339), and BSC (SUCRA 0.141). Compared to chemotherapy, multi-targeted therapy (HR 0.57, 95% CI 0.37–0.87) and targeted therapy plus chemotherapy (HR 0.78, 95% CI 0.67–0.91) had significant advantages. Targeted therapy (HR 0.92, 95% CI 0.54–1.57) and local treatment plus chemotherapy (HR 1.03, 95% CI 0.85–1.23) showed consistent efficacy, when compared to chemotherapy. Targeted therapy plus chemotherapy was superior. Among them, aflibercept plus CTFU showed the best performance. In comparison to aflibercept plus CTFU, the performance ranked from good to poor were bevacizumab plus CTCA (HR 1.04, 95% CI 0.68–1.6), bevacizumab plus CTFU (HR 1.23, 95% CI 0.85–1.77), panitumumab plus CTFU (HR 1.31, 95% CI 0.9–1.91), and simvastatin plus CTFU (HR 1.41, 95% CI 0.88–2.26). There were no significant differences observed among targeted or local treatment plus chemotherapy regimens. Among local treatment, HAI had the best performance, followed by TACE and TARE. Among target therapies, fruquintinib had the best efficacy. Compared to fruquintinib, the treatments with no significant difference, ordered from best to worst, were regorafenib (HR 1.03, 95% CI 0.7–1.53), TAS-102 (HR 1.14, 95% CI 0.77–1.68), cetuximab (HR 1.19, 95% CI 0.48–2.9), and panitumumab (HR 1.41, 95% CI 0.65–3.05). Comparatively, anlotinib (HR 1.58, 95% CI 1.16–2.16), nintedanib (HR 2.29, 95% CI 1.64–3.19), ramucirumab (HR 1.66, 95% CI 1.12–2.44), and napabucasin (HR 2.29, 95% CI 1.64–3.19) showed significant differences in efficacy compared to fruquintinib. Furthermore, CTCA (HR 0.66, 95% CI 0.2–2.18) had better efficacy compared to CTFU. More details, please see **Tables 2**, **3** and **Figure 3**.

**Table 2:** Overall survival profiles of treatment regimens in metastatic CRC patients with liver metastases and multiple-site metastases.

OS for Liver metastases	OS for Multiple-site Metastases																				
	AFI + CTFU	NA	0.79 (0.57, 1.1)	0.93 (0.72, 1.19)	1.67 (0.86, 3.27)	1.02 (0.47, 2.22)	0.99 (0.51, 1.96)	**1.21 (1.02, 1.44)**	1.02 (0.5, 2.07)	NA	2.21 (0.97, 5.02)	1.45 (0.73, 2.91)	0.99 (0.46, 2.1)	1.03 (0.81, 1.32)	1.43 (0.7, 2.91)	1.01 (0.47, 2.15)	NA	NA	NA	1.11 (0.63, 1.95)	0.68 (0.42, 1.09)
	0.49 (0.23, 1.06)	ANL	NA	NA	NA	NA	NA	NA	NA	NA	NA	NA	NA	NA	NA	NA	NA	NA	NA	NA	NA
	0.96 (0.62, 1.48)	1.94 (0.96, 3.95)	BEV + CTCA	1.18 (0.95, 1.45)	**2.12 (1.1, 4.08)**	1.29 (0.6, 2.77)	1.26 (0.62, 2.56)	**1.53 (1.16, 2.02)**	1.29 (0.64, 2.59)	NA	**2.79 (1.24, 6.26)**	1.84 (0.93, 3.63)	1.25 (0.59, 2.62)	1.31 (0.96, 1.78)	1.81 (0.9, 3.64)	1.27 (0.6, 2.68)	NA	NA	NA	1.4 (0.8, 2.43)	0.86 (0.55, 1.35)
	0.82 (0.56, 1.18)	1.65 (0.84, 3.24)	0.85 (0.68, 1.06)	BEV + CTFU	1.8 (0.97, 3.35)	1.09 (0.52, 2.29)	1.07 (0.54, 2.1)	**1.3 (1.09, 1.56)**	1.1 (0.56, 2.13)	NA	**2.37 (1.09, 5.18)**	1.57 (0.82, 2.98)	1.06 (0.52, 2.16)	1.11 (0.88, 1.4)	1.54 (0.79, 2.99)	1.08 (0.53, 2.22)	NA	NA	NA	1.19 (0.71, 1.98)	0.73 (0.49, 1.09)
	**0.45 (0.22, 0.94)**	0.92 (0.71, 1.2)	**0.47 (0.25, 0.92)**	0.56 (0.3, 1.04)	BSC	**0.61 (0.41, 0.91)**	0.59 (0.24, 1.48)	0.72 (0.38, 1.38)	**0.61 (0.48, 0.78)**	NA	1.32 (0.82, 2.11)	0.87 (0.73, 1.03)	**0.59 (0.42, 0.84)**	0.62 (0.32, 1.19)	0.85 (0.67, 1.08)	**0.6 (0.42, 0.86)**	NA	NA	NA	**0.66 (0.46, 0.94)**	**0.41 (0.25, 0.65)**
	0.66 (0.21, 2.07)	1.33 (0.53, 3.36)	0.69 (0.23, 2.08)	0.81 (0.27, 2.39)	1.45 (0.6, 3.52)	CET	0.98 (0.36, 2.66)	1.19 (0.56, 2.55)	1 (0.63, 1.61)	NA	**2.17 (1.17, 4.04)**	1.43 (0.92, 2.22)	0.97 (0.79, 1.19)	1.02 (0.47, 2.2)	1.41 (0.88, 2.24)	0.99 (0.57, 1.69)	NA	NA	NA	1.09 (0.64, 1.85)	0.67 (0.36, 1.24)
	0.98 (0.29, 3.36)	1.99 (0.49, 8.07)	1.02 (0.3, 3.56)	1.21 (0.36, 4.09)	2.17 (0.55, 8.55)	1.5 (0.29, 7.71)	CTCA	1.22 (0.63, 2.35)	1.03 (0.4, 2.65)	NA	2.22 (0.8, 6.25)	1.47 (0.58, 3.73)	0.99 (0.37, 2.65)	1.04 (0.53, 2.04)	1.44 (0.56, 3.7)	1.01 (0.38, 2.71)	NA	NA	NA	1.11 (0.48, 2.59)	0.68 (0.31, 1.5)
	**0.65 (0.49, 0.86)**	1.32 (0.64, 2.69)	**0.68 (0.49, 0.94)**	0.8 (0.62, 1.02)	1.43 (0.73, 2.78)	0.99 (0.32, 2.98)	0.66 (0.2, 2.18)	CTFU	0.84 (0.42, 1.68)	NA	1.82 (0.82, 4.05)	1.2 (0.62, 2.35)	0.81 (0.39, 1.7)	0.85 (0.72, 1.01)	1.18 (0.6, 2.35)	0.83 (0.4, 1.74)	NA	NA	NA	0.91 (0.53, 1.56)	**0.56 (0.36, 0.87)**
	0.78 (0.37, 1.64)	**1.58 (1.16, 2.16)**	0.81 (0.41, 1.61)	0.96 (0.5, 1.83)	**1.72 (1.46, 2.03)**	1.19 (0.48, 2.9)	0.79 (0.2, 3.17)	1.2 (0.6, 2.4)	FRU	NA	**2.16 (1.27, 3.67)**	**1.43 (1.06, 1.92)**	0.97 (0.63, 1.48)	1.01 (0.5, 2.04)	**1.4 (1, 1.97)**	0.98 (0.64, 1.52)	NA	NA	NA	1.08 (0.71, 1.66)	0.67 (0.39, 1.13)
	1.14 (0.25, 5.19)	2.32 (0.45, 12.02)	1.19 (0.26, 5.44)	1.4 (0.31, 6.28)	2.52 (0.49, 12.76)	1.74 (0.27, 11.09)	1.16 (0.49, 2.76)	1.76 (0.4, 7.79)	1.47 (0.28, 7.49)	HAI + SCT	NA	NA	NA	NA	NA	NA	NA	NA	NA	NA	NA
	**0.34 (0.16, 0.75)**	0.69 (0.47, 1.02)	**0.36 (0.17, 0.73)**	**0.42 (0.21, 0.83)**	**0.75 (0.56, 1)**	0.52 (0.2, 1.32)	0.35 (0.09, 1.42)	0.53 (0.25, 1.09)	**0.44 (0.31, 0.61)**	0.3 (0.06, 1.56)	NAP	0.66 (0.4, 1.09)	**0.45 (0.25, 0.8)**	0.47 (0.21, 1.05)	0.65 (0.38, 1.1)	**0.45 (0.25, 0.82)**	NA	NA	NA	**0.5 (0.28, 0.9)**	**0.31 (0.16, 0.6)**
	0.48 (0.23, 1.01)	0.97 (0.7, 1.34)	**0.5 (0.25, 0.99)**	0.59 (0.31, 1.12)	1.05 (0.88, 1.26)	0.73 (0.29, 1.78)	0.49 (0.12, 1.95)	0.74 (0.37, 1.47)	**0.61 (0.48, 0.78)**	0.42 (0.08, 2.15)	**1.4 (1, 1.97)**	NIN	0.68 (0.46, 1)	0.71 (0.36, 1.4)	0.98 (0.73, 1.32)	0.69 (0.46, 1.03)	NA	NA	NA	0.76 (0.51, 1.12)	**0.47 (0.28, 0.77)**
	0.55 (0.2, 1.58)	1.12 (0.51, 2.49)	0.58 (0.21, 1.57)	0.68 (0.26, 1.81)	1.22 (0.57, 2.59)	0.84 (0.53, 1.34)	0.56 (0.12, 2.71)	0.85 (0.31, 2.34)	0.71 (0.33, 1.53)	0.48 (0.08, 2.93)	1.62 (0.72, 3.63)	1.16 (0.53, 2.51)	PAN	1.05 (0.5, 2.21)	1.45 (0.95, 2.2)	1.02 (0.62, 1.68)	NA	NA	NA	1.12 (0.68, 1.83)	0.69 (0.38, 1.23)
	0.76 (0.52, 1.11)	1.55 (0.75, 3.19)	0.8 (0.56, 1.12)	0.94 (0.72, 1.22)	1.68 (0.85, 3.29)	1.16 (0.38, 3.51)	0.78 (0.23, 2.65)	1.17 (0.91, 1.52)	0.98 (0.48, 1.96)	0.67 (0.15, 3.01)	**2.24 (1.07, 4.65)**	1.6 (0.79, 3.21)	1.38 (0.5, 3.79)	PAN + CTFU	1.38 (0.69, 2.79)	0.97 (0.46, 2.06)	NA	NA	NA	1.07 (0.61, 1.87)	0.66 (0.41, 1.04)
	0.47 (0.21, 1.05)	0.96 (0.62, 1.48)	0.49 (0.23, 1.04)	0.58 (0.28, 1.18)	1.04 (0.73, 1.48)	0.72 (0.28, 1.85)	0.48 (0.12, 1.98)	0.73 (0.34, 1.54)	**0.6 (0.41, 0.89)**	0.41 (0.08, 2.18)	1.38 (0.88, 2.17)	0.99 (0.66, 1.47)	0.85 (0.37, 1.95)	0.62 (0.29, 1.32)	RAM	0.7 (0.46, 1.08)	NA	NA	NA	0.77 (0.51, 1.18)	**0.47 (0.28, 0.8)**
	0.76 (0.34, 1.7)	1.53 (0.98, 2.39)	0.79 (0.37, 1.67)	0.93 (0.45, 1.9)	**1.67 (1.16, 2.38)**	1.15 (0.44, 2.98)	0.77 (0.19, 3.2)	1.16 (0.55, 2.49)	0.97 (0.65, 1.44)	0.66 (0.13, 3.51)	**2.21 (1.4, 3.51)**	**1.58 (1.06, 2.36)**	1.37 (0.59, 3.14)	0.99 (0.46, 2.14)	1.6 (0.97, 2.65)	REG	NA	NA	NA	1.1 (0.67, 1.82)	0.68 (0.37, 1.22)
	0.71 (0.44, 1.14)	1.44 (0.64, 3.25)	0.74 (0.45, 1.23)	0.87 (0.55, 1.38)	1.57 (0.72, 3.38)	1.08 (0.33, 3.47)	0.72 (0.2, 2.54)	1.09 (0.74, 1.61)	0.91 (0.41, 2)	0.62 (0.13, 2.88)	2.08 (0.91, 4.74)	1.49 (0.67, 3.28)	1.28 (0.44, 3.77)	0.93 (0.59, 1.48)	1.51 (0.65, 3.51)	0.94 (0.4, 2.2)	SIM + CTFU	NA	NA	NA	NA
	0.71 (0.44, 1.14)	1.43 (0.63, 3.24)	0.74 (0.44, 1.23)	0.86 (0.54, 1.37)	1.56 (0.71, 3.37)	1.07 (0.33, 3.47)	0.72 (0.2, 2.53)	1.09 (0.73, 1.61)	0.9 (0.41, 1.99)	0.62 (0.13, 2.87)	2.07 (0.9, 4.71)	1.48 (0.66, 3.27)	1.28 (0.43, 3.75)	0.93 (0.58, 1.48)	1.5 (0.64, 3.49)	0.94 (0.4, 2.18)	0.99 (0.57, 1.73)	TACE + CTFU	NA	NA	NA
	**0.61 (0.43, 0.86)**	1.23 (0.58, 2.6)	**0.63 (0.43, 0.94)**	0.74 (0.54, 1.03)	1.34 (0.66, 2.69)	0.92 (0.3, 2.84)	0.62 (0.18, 2.08)	0.93 (0.75, 1.16)	0.78 (0.38, 1.6)	0.53 (0.12, 2.39)	1.78 (0.83, 3.79)	1.27 (0.61, 2.62)	1.1 (0.39, 3.05)	0.8 (0.57, 1.11)	1.29 (0.59, 2.81)	0.8 (0.36, 1.76)	0.85 (0.55, 1.33)	0.86 (0.55, 1.35)	TARE + CTFU	NA	NA
	0.69 (0.37, 1.29)	1.39 (0.9, 2.16)	0.72 (0.41, 1.25)	0.84 (0.51, 1.41)	**1.51 (1.06, 2.15)**	1.04 (0.4, 2.69)	**0.7 (0.19, 2.63)**	1.06 (0.6, 1.87)	0.88 (0.6, 1.3)	0.6 (0.12, 2.95)	**2.01 (1.28, 3.18)**	1.44 (0.97, 2.14)	1.24 (0.54, 2.85)	0.9 (0.51, 1.61)	1.46 (0.89, 2.39)	0.91 (0.55, 1.5)	0.97 (0.49, 1.92)	0.97 (0.49, 1.95)	1.13 (0.62, 2.08)	TAS 102	**0.61 (0.45, 0.84)**
	1.12 (0.65, 1.93)	**2.27 (1.32, 3.89)**	1.17 (0.74, 1.85)	1.37 (0.92, 2.05)	**2.47 (1.53, 3.95)**	1.7 (0.62, 4.63)	1.14 (0.31, 4.13)	**1.72 (1.08, 2.76)**	1.43 (0.87, 2.36)	0.98 (0.21, 4.63)	**3.28 (1.88, 5.7)**	**2.34 (1.41, 3.88)**	2.02 (0.83, 4.91)	1.47 (0.91, 2.38)	**2.37 (1.32, 4.26)**	1.48 (0.82, 2.68)	1.58 (0.86, 2.9)	1.59 (0.86, 2.93)	**1.84 (1.1, 3.09)**	**1.63 (1.19, 2.23)**	TAS 102 + BEV

AFI + CTFU, Aflibercept plus CTFU; ANL, Anlotinib; BEV + CTCA, Bevacizumab plus CTCA; BEV + CTFU, Bevacizumab plus CTFU; BSC, best supportive care; CET, Cetuximab; CTCA, capecitabine-based combination chemotherapy; CTFU, fluorouracil-based combination chemotherapy; FRU, Fruquintinib; HAI + SCT, HAI followed by SingleCT; NAP, Napabucasin; NIN, Nintedanib; PAN, Panitumumab; PAN + CTFU, Panitumumab plus CTFU; RAM, Ramucirumab; REG, Regorafenib; SIM + CTFU, Simvastatin plus CTFU; TAS 102, TAS-102; TAS 102 + BEV, TAS-102 plus bevacizumab; TACE, transarterial chemoembolization; TARE, transarterial radioembolization. Values in blue background indicate significant differences between schemes, while those in other colors indicate no significant differences. Values in bold also indicate statistically significant differences. NA, not available.

**Table 3:** Overall survival profiles of the treatment mechanisms in patients with liver metastases.

BSC (SUCRA 0.141)					
1.16 (0.67, 1.99)	CT (SUCRA 0.339)				
1.13 (0.64, 2)	0.98 (0.81, 1.17)	Local treatment plus CT (SUCRA 0.286)			
**2.04 (1.47, 2.83)**	**1.76 (1.15, 2.71)**	**1.81 (1.13, 2.89)**	Multi-Targeted (SUCRA 0.985)		
**1.25 (1.14, 1.38)**	1.08 (0.64, 1.84)	1.11 (0.63, 1.95)	**0.61 (0.45, 0.84)**	Targeted (SUCRA 0.503)	
1.49 (0.89, 2.5)	**1.29 (1.1, 1.5)**	**1.32 (1.04, 1.68)**	0.73 (0.49, 1.09)	1.19 (0.72, 1.98)	Targeted plus CT (SUCRA 0.746)

BSC, best supportive care; CT, chemotherapy; Targeted, Targeted therapy. Values in blue background indicate significant differences between schemes, while those in other colors indicate no significant differences. Values in bold also indicate statistically significant differences.

**Figure 4:**
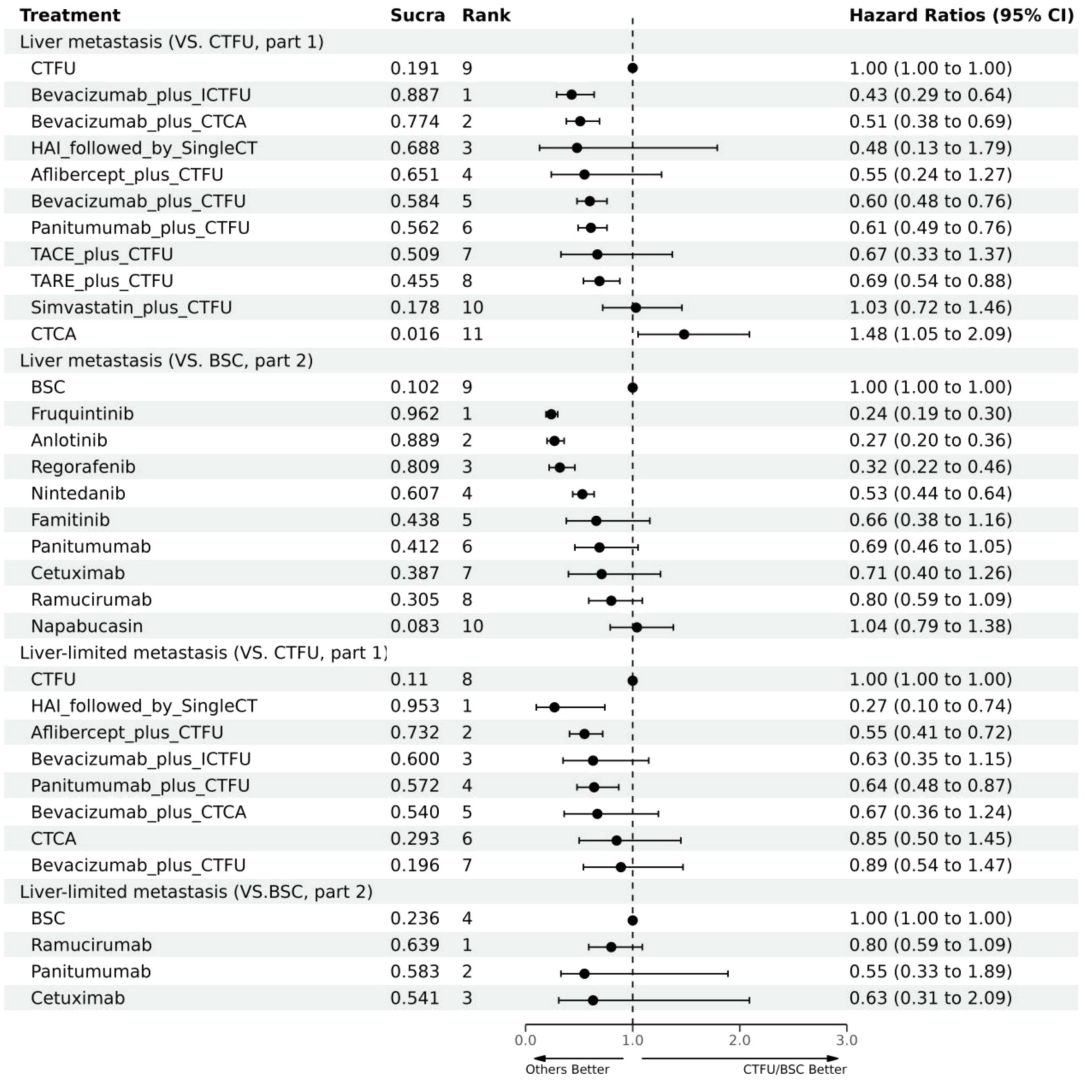
Forest plot for PFS. CTFU, fluorouracil-based combination chemotherapy; CTCA, capecitabine-based combination chemotherapy; ICTFU, intensified chemotherapy containing four interventions; SingleCT, single-agent chemotherapy.

For PFS, due to the lack of sufficient data, we can only analyze monotherapy and combination therapies separately. Network plots is shown in **Figures 2F, G**. For combination therapies, the top three ranked regimes were bevacizumab plus ICTFU (SUCRA 0.887), bevacizumab plus CTCA (SUCRA 0.774), HAI followed by SingleCT (SUCRA 0.688). Targeted combination chemotherapy shows significant advantages over CTFU, specifically, the rankings from high to low were bevacizumab plus ICTFU (HR 0.43, 95% CI 0.29–0.64), bevacizumab plus CTCA (HR 0.51, 95% CI 0.38–0.69), bevacizumab plus CTFU (HR 0.6, 95% CI 0.48–0.76), panitumumab plus CTFU (HR 0.61, 95% CI 0.49–0.76). Overall, targeted therapy plus chemotherapy had the better efficacy than local treatment plus chemotherapy and chemotherapy; the effects of local treatment combination chemotherapy and chemotherapy tended to be consistent. CTFU performed better than CTCA. In monotherapies, the top three ranked were fruquintinib (SUCRA 0.962), anlotinib (SUCRA 0.889), and regorafenib (SUCRA 0.809). Compared to BSC, treatments with significant advantages were fruquintinib (HR 0.24, 95% CI 0.19–0.3), anlotinib (HR 0.27, 95% CI 0.2–0.36), regorafenib (HR 0.32, 95% CI 0.22–0.46), nintedanib (HR 0.53, 95% CI 0.44–0.64). Full information is available in **Tables 4**, **5** and **Figure 4**.

**Table 4:** Progression-free survival profiles of monotherapy regimens in metastatic CRC patients with liver metastases and liver-limited metastases.

PFS for Liver metastases	PFS for liver-limited metastases									
	ANL	NA	NA	NA	NA	NA	NA	NA	NA	NA
	**0.27 (0.2, 0.36)**	BSC	0.81 (0.31, 2.09)	NA	NA	NA	NA	0.79 (0.33, 1.89)	0.8 (0.59, 1.09)	NA
	**0.38 (0.2, 0.73)**	1.41 (0.79, 2.5)	CET	NA	NA	NA	NA	0.98 (0.66, 1.46)	0.99 (0.36, 2.7)	NA
	**0.41 (0.22, 0.77)**	1.51 (0.86, 2.65)	1.07 (0.48, 2.4)	FAM	NA	NA	NA	NA	NA	NA
	1.14 (0.78, 1.68)	**4.23 (3.31, 5.4)**	**3 (1.61, 5.6)**	**2.8 (1.52, 5.16)**	FRU	NA	NA	NA	NA	NA
	**0.26 (0.17, 0.39)**	0.96 (0.73, 1.27)	0.68 (0.36, 1.29)	0.64 (0.34, 1.19)	**0.23 (0.16, 0.33)**	NAP	NA	NA	NA	NA
	**0.51 (0.36, 0.72)**	**1.89 (1.57, 2.28)**	1.34 (0.73, 2.45)	1.25 (0.69, 2.25)	**0.45 (0.33, 0.61)**	**1.96 (1.4, 2.75)**	NIN	NA	NA	NA
	**0.39 (0.23, 0.65)**	1.44 (0.95, 2.17)	1.02 (0.68, 1.52)	0.95 (0.47, 1.91)	**0.34 (0.21, 0.55)**	1.5 (0.91, 2.46)	0.76 (0.48, 1.2)	PAN	1.01 (0.4, 2.54)	NA
	**0.34 (0.22, 0.52)**	1.25 (0.92, 1.7)	0.88 (0.46, 1.7)	0.82 (0.44, 1.56)	**0.3 (0.2, 0.44)**	1.3 (0.86, 1.96)	**0.66 (0.46, 0.95)**	0.87 (0.52, 1.45)	RAM	NA
	0.84 (0.53, 1.35)	**3.13 (2.16, 4.51)**	**2.21 (1.12, 4.38)**	**2.06 (1.06, 4.03)**	0.74 (0.47, 1.15)	**3.25 (2.05, 5.15)**	**1.66 (1.09, 2.5)**	**2.17 (1.25, 3.77)**	**2.5 (1.55, 4.04)**	REG

ANL, Anlotinib; BSC, best supportive care; CET, Cetuximab; FAM, Famitinib; FRU, Fruquintinib; NAP, Napabucasin; NIN, Nintedanib; PAN, Panitumumab; RAM, Ramucirumab; REG, Regorafenib. Values in blue background indicate significant differences between schemes, while those in other colors indicate no significant differences. Values in bold also indicate statistically significant differences. NA, not available.

**Table 5:** Progression-free survival profiles of combined therapies in metastatic CRC patients with liver metastases and liver-limited metastases.

PFS for Liver metastases	PFS for liver-limited metastases										
	AFI+CTFU	1.22 (0.62, 2.41)	1.63 (0.92, 2.88)	1.15 (0.6, 2.23)	1.55 (0.85, 2.86)	**1.83 (1.38, 2.42)**	0.5 (0.18, 1.41)	1.18 (0.78, 1.77)	NA	NA	NA
	1.07 (0.44, 2.62)	BEV+CTCA	1.33 (0.92, 1.93)	0.95 (0.58, 1.55)	1.28 (0.56, 2.9)	1.5 (0.81, 2.8)	0.41 (0.13, 1.33)	0.97 (0.53, 1.78)	NA	NA	NA
	0.91 (0.38, 2.17)	0.85 (0.69, 1.04)	BEV+CTFU	**0.71 (0.51, 0.98)**	0.96 (0.46, 1.99)	1.12 (0.68, 1.85)	**0.31 (0.1, 0.94)**	0.72 (0.44, 1.18)	NA	NA	NA
	1.28 (0.51, 3.24)	1.2 (0.81, 1.76)	**1.41 (1.02, 1.95)**	BEV+ICTFU	1.35 (0.6, 3)	1.58 (0.87, 2.87)	0.43 (0.13, 1.38)	1.02 (0.57, 1.83)	NA	NA	NA
	**0.37 (0.15, 0.91)**	**0.35 (0.22, 0.55)**	**0.41 (0.27, 0.61)**	**0.29 (0.17, 0.49)**	CTCA	1.18 (0.69, 2.01)	**0.32 (0.14, 0.75)**	0.76 (0.41, 1.4)	NA	NA	NA
	0.55 (0.24, 1.27)	**0.51 (0.38, 0.69)**	**0.6 (0.48, 0.76)**	**0.43 (0.29, 0.64)**	**1.48 (1.05, 2.09)**	CTFU	**0.27 (0.1, 0.74)**	**0.64 (0.48, 0.87)**	NA	NA	NA
	1.15 (0.24, 5.52)	1.07 (0.28, 4.21)	1.26 (0.33, 4.86)	0.9 (0.23, 3.58)	3.11 (0.86, 11.21)	2.1 (0.56, 7.91)	HAI+SCT	2.36 (0.83, 6.74)	NA	NA	NA
	0.9 (0.38, 2.15)	0.84 (0.67, 1.06)	0.99 (0.88, 1.1)	0.7 (0.5, 0.99)	**2.43 (1.62, 3.66)**	**1.64 (1.31, 2.05)**	0.78 (0.2, 2.98)	PAN+CTFU	NA	NA	NA
	0.53 (0.21, 1.32)	**0.5 (0.31, 0.79)**	**0.58 (0.39, 0.89)**	**0.42 (0.24, 0.71)**	1.44 (0.88, 2.36)	0.97 (0.68, 1.38)	0.46 (0.12, 1.82)	**0.59 (0.39, 0.9)**	SIM+CTFU	NA	NA
	0.82 (0.27, 2.46)	0.76 (0.35, 1.67)	0.9 (0.42, 1.9)	0.64 (0.28, 1.45)	**2.21 (1, 4.89)**	1.49 (0.73, 3.05)	0.71 (0.16, 3.19)	0.91 (0.43, 1.92)	1.54 (0.69, 3.41)	TACE+CTFU	NA
	0.79 (0.33, 1.9)	0.74 (0.5, 1.09)	0.87 (0.63, 1.22)	**0.62 (0.39, 0.99)**	**2.15 (1.41, 3.27)**	**1.45 (1.13, 1.85)**	0.69 (0.18, 2.64)	0.88 (0.63, 1.23)	1.49 (0.97, 2.3)	0.97 (0.46, 2.07)	TARE+CTFU

AFI + CTFU, Aflibercept plus CTFU; BEV + CTCA, Bevacizumab plus CTCA; BEV + CTFU, Bevacizumab plus CTFU; BEV+ICTFU, Bevacizumab plus ICTFU; BSC, best supportive care; CET, Cetuximab; CTCA, capecitabine-based combination chemotherapy; CTFU, fluorouracil-based combination chemotherapy; HAI + SCT, HAI followed by SingleCT; PAN + CTFU, Panitumumab plus CTFU; SIM + CTFU, Simvastatin plus CTFU; TACE, transarterial chemoembolization; TARE, transarterial radioembolization. Values in blue background indicate significant differences between schemes, while those in other colors indicate no significant differences. Values in bold also indicate statistically significant differences. NA, not available.

#### 3.3.2 Liver-limited metastases

In terms of OS, similarly, due to data insufficiency, we divided this section into two parts for analysis: In the combination therapy, compared to CTFU, only aflibercept plus CTFU (HR 0.65, 95% CI 0.49–0.86) showed the significant advantage. Meanwhile, there was no significant difference in efficacy between the combination therapies. For monotherapy, cetuximab (HR 0.69, 95% CI 0.29–1.66), panitumumab (HR 0.82, 95% CI 0.39–1.74), ramucirumab (HR 0.96, 95% CI 0.68–1.37) were all superior to BSC, but the advantages were not statistically significant. Network plots is shown in **Figure 2D, E**, forest plots and league table are provided in **Figure 3** and **Table 6**.

**Table 6:** Overall survival profiles of combined therapies (A) and monotherapy regimens (B) in metastatic CRC patients with liver-limited metastases.

A						
AFI+CTFU
0.82 (0.45, 1.49)	BEV+CTCA
0.82 (0.56, 1.2)	1 (0.63, 1.6)	BEV+CTFU
0.98 (0.29, 3.34)	1.2 (0.32, 4.45)	1.2 (0.35, 4.08)	CTCA
**0.65 (0.49, 0.86)**	0.79 (0.46, 1.36)	0.8 (0.61, 1.03)	0.66 (0.2, 2.19)	CTFU
1.14 (0.25, 5.12)	1.39 (0.29, 6.75)	1.39 (0.31, 6.26)	1.16 (0.49, 2.76)	1.75 (0.4, 7.71)	HAI+SCT
0.76 (0.51, 1.13)	0.93 (0.52, 1.66)	0.93 (0.67, 1.31)	0.78 (0.23, 2.65)	1.17 (0.89, 1.55)	0.67 (0.15, 3)	PAN+CTFU
B						
BSC	
1.45 (0.6, 3.46)		Cetuximab	
1.22 (0.58, 2.56)		0.84 (0.53, 1.34)		Panitumumab	
1.04 (0.73, 1.47)		0.72 (0.28, 1.85)		0.85 (0.37, 1.95)		Ramucirumab

AFI + CTFU, Aflibercept plus CTFU; BEV + CTCA, Bevacizumab plus CTCA; BEV + CTFU, Bevacizumab plus CTFU; BSC, best supportive care; CTCA, capecitabine-based combination chemotherapy; CTFU, fluorouracil-based combination chemotherapy; HAI + SCT, HAI followed by SingleCT; PAN + CTFU, Panitumumab plus CTFU. Values in blue background indicate significant differences between schemes, while those in other colors indicate no significant differences. Values in bold also indicate statistically significant differences.

In terms of PFS, the top three rankings in combination therapy were as follows: HAI followed by SingleCT (SUCRA 0.953), aflibercept plus CTFU (SUCRA 0.732), and bevacizumab plus ICTFU (SUCRA 0.6). Compared to CTFU, the ones with significant advantages were HAI followed by SingleCT (HR 0.27, 95% CI 0.1–0.74), aflibercept plus CTFU (HR 0.55, 95% CI 0.41–0.72), and panitumumab plus CTFU (HR 0.64, 95% CI 0.48–0.87). Among the monotherapy options, ramucirumab performed the best. However, compared to BSC, ramucirumab (HR 0.8, 95% CI 0.59–1.09), panitumumab (HR 0.55, 95% CI 0.33–1.89), and cetuximab (HR 0.63, 95% CI 0.31–2.09) did not demonstrate significant advantages. Other information is presented in **Figures 2H, I**, **4** and **Tables 4**, **5**.

#### 3.3.3 Multiple-site metastases

Due to insufficient data, this section only considered OS. Network plot is provided as **Figure 2C**. The top three rankings were as follows: TAS-102 plus bevacizumab (SUCRA 0.936), bevacizumab plus CTCA (SUCRA 0.836), and bevacizumab plus CTFU (SUCRA 0.672). Compared to CTFU, the strategies with significant advantages were TAS-102 plus bevacizumab (HR 0.56, 95% CI 0.36–0.87), bevacizumab plus CTCA (HR 0.65, 95% CI 0.5–0.86), bevacizumab plus CTFU (HR 0.77, 95% CI 0.64–0.92), and aflibercept plus CTFU (HR 0.83, 95% CI 0.69–0.98). The efficacy of multi-targeted therapy and targeted therapy plus chemotherapy regimens were superior to targeted therapy and chemotherapy. Among targeted therapy plus chemotherapy regimens, bevacizumab plus CTCA or CTFU performed the best, followed by aflibercept plus CTFU and panitumumab plus CTFU, although there was no significant difference in efficacy between these regimens. For monotherapies, panitumumab (HR 0.81, 95% CI 0.39–1.7), regorafenib (HR 0.83, 95% CI 0.4–1.74), fruquintinib (HR 0.84, 95% CI 0.42–1.68), cetuximab (HR 0.84, 95% CI 0.39–1.8), and TAS-102 (HR 0.91, 95% CI 0.53–1.56) had advantages over CTFU, although without statistical significance. Ramucirumab (HR 1.18, 95% CI 0.6–2.35), nintedanib (HR 1.2, 95% CI 0.62–2.35), and napabucasin (HR 1.82, 95% CI 0.82–4.05) performed worse compared to CTFU. More details are provided in **Figure 3** and **Table 2**.

### 3.4 Heterogeneity analysis

The results of the heterogeneity test are summarized in **Supplementary File 5**. It was observed that there was low heterogeneity in almost all of the comparisons. However, high heterogeneity was only detected in targeted therapy VS. BSC (77.2%) in the mechanism comparison.

## 4 Discussions

This NMA systematically compared the efficacy of treatment options for patients with unresectable CRLM who have not responded to at least one prior line of previous systemic therapy. The main findings of this study are summarized as follows:

1. In comparison to chemotherapy, multi-targeted therapy (HR 0.57, 95% CI 0.37–0.87) and targeted therapy plus chemotherapy (HR 0.78, 95% CI 0.67–0.91) show significant advantages; Targeted therapy (HR 0.92, 95% CI 0.54–1.57) and local treatment plus chemotherapy (HR 1.03, 95% CI 0.85–1.23) had comparable performance.
2. For patients with liver metastases, TAS-102 plus bevacizumab, aflibercept plus CTFU, and bevacizumab plus CTCA showed the best outcomes in terms of OS. Bevacizumab plus ICTFU, bevacizumab plus CTCA, and HAI followed by SingleCT performed the best in terms of PFS.
3. For patients with liver-limited metastases, aflibercept plus CTFU was the optimal choice for OS. In terms of PFS, the best options were HAI followed by SingleCT, aflibercept plus CTFU, and panitumumab plus CTFU.
4. For patients with multiple-site metastases, the best treatments were TAS-102 plus bevacizumab, bevacizumab plus CTCA, bevacizumab plus CTFU, and aflibercept plus CTFU.

The use of multi-targeted therapies, such as TAS-102 plus bevacizumab, showed significant effectiveness in prolonging the OS. TAS-102 is an orally active and well tolerated drug composed of trifluridine and tipiracil. Trifluridine, the active antitumor component, is phosphorylated by thymidine kinase within cancer cells to produce trifluridine triphosphate. This trifluridine triphosphate is then substituted for thymidine in DNA. Although the precise mechanism of action for bevacizumab is not fully understood, it likely normalizes tumor blood vessel structure, thereby boosting the blood supply to the tumor. By combining bevacizumab with TAS-102, there is a possibility of elevating trifluridine concentrations specifically within tumor DNA without causing increased overall systemic exposure or toxicity of trifluridine. At the same time, previously treated patients with metastatic CRC exhibit acceptable toxicity when treated with TAS-102 and bevacizumab together (34). Cetuximab, an EGFR inhibitor, showed a strong correlation with increased tumor response. Moreover, it expedited symptom relief in patients with responsive tumors, providing additional benefits (62). Panitumumab, another EGFR inhibitor, is the standard treatment for patients with wild-type metastatic CRC. Studies have shown that anti-EGFR therapy can induce tumor-specific adaptive immune responses and immunogenic cell death. Aflibercept is also a VEGF inhibitor, similar to bevacizumab. It is a recombinant fusion protein that includes parts of human VEGF receptors 1 and 2, fused to the Fc portion of human immunoglobulin G1. This fusion protein blocks the activity of VEGFA, VEGFB, and placental growth factor by acting as a high-affinity ligand trap, preventing these ligands from binding to their natural receptors. Therefore, combined anti-angiogenesis therapies with chemotherapy can further improve the patients’ survival (63).

Currently, there have been no studies that have conducted a systematic evaluation of treatment options for previously treated liver metastases in patients with CRC. There are only a few articles that have conducted a meta-analysis on the partial treatments for unresectable CRLM, but the quality and quantity of the included trials were insufficient. Sanne et al. compared ablation, irreversible electroporation, and stereotactic ablative body radiotherapy (SABR) for unresectable CRLM, while no RCTs were included (64). The control rates varied between 22% and 90% for all techniques; thermal ablation had a range of 22% to 89%, irreversible electroporation had 44%, and SABR had a range of 67% to 90% depending on the radiation dose. Focal ablative therapy was a safe option that can lead to long-term disease control. Simone et al. conducted a comparison between HAI and systemic chemotherapy for unresectable CRLM (65). They discovered that the tumor response rate was 42.9% for HAI and 18.4% for systemic chemotherapy. However, no significant difference in the meta-risk of death was observed between the two treatments. Jordan et al. studied on intra-arterial treatments for unresectable and chemorefractory CRLM. They discovered that the combined response rates for cTACE, drug-eluting embolic TACE (DEE-TACE), and TARE were 23%, 36%, and 23% respectively (66). The median survival times and ranges for cTACE, DEB-TACE, and TARE were 16 months (9–23), 16 months (7–25), and 12 months (7–15) respectively. Daniel et al. examined how HAI treatment could potentially be used as a neoadjuvant therapy performing hepatic resection. They discovered that 50% of patients responded positively to HAI, and 18% of patients were able to undergo surgery afterwards (67). Joseph et al. assessed the effectiveness of HAI, cTACE, DEE-TACE, and TARE, in combinations with systemic chemotherapy, for treating unresectable CRLM (68). They discovered that the combination of DEE-TACE and systemic chemotherapy yielded the most favorable oncological results and had the greatest potential for conversion to resection. Cardiovascular complications, including arrhythmias, hypertension, and more severe outcomes such as heart failure and myocardial infarction, are particularly pertinent due to the high vascular nature of the liver and the intense metabolic demands of metastatic cancer. The impact of these cardiovascular side events on overall survival is significant, as they can limit treatment efficacy and adversely affect patient quality of life. In the patents discussed, polydatin, a nutraceutical derived from Polygonum cuspidatum, is highlighted for its potential to enhance the effectiveness of anticancer drugs such as 5-fluorouracil, cisplatin, and tyrosine kinase inhibitors. Polydatin has shown cardioprotective effects and the ability to boost the efficacy of these treatments in clinical studies. Its dual role in reducing cardiovascular side effects and improving treatment outcomes offers a promising avenue for integrated cancer therapies (69).

This study conducted the first systematic evaluation and NMA of the effectiveness of different strategies in previously treated CRLM patients. This has significant implications for the development of clinical medications and related guidelines. In contrast to previous studies, this research meticulously grouped interventions based on the type and mechanism of chemotherapy to minimize heterogeneity to the greatest extent. Moreover, the heterogeneity of this study’s results was low, indicating that the conclusions of this study are relatively reliable. Additionally, we performed multiple subgroup analyses, which hold extraordinary significance for the precise treatment of CRC. We differentiated the analysis between patients with liver-limited metastases and those with multiple-site metastases, providing more evidence support for clinical diagnosis and treatment decisions. After a systematic comparison, the viewpoints we propose are innovative and deserve further discussion, as they will also provide new directions for future clinical research.

Due to data availability, this study has some limitations. The majority of included RCTs did not report safety data for CRLM patients, therefore, we could not systematically evaluate the safety. For the same reason, we were unable to analyze patients with biomarker mutations; Furthermore, due to lack of individual data and most studies only reporting HR, we had to use the Cox proportional hazards model for indirect comparison, instead of other risk-varying models. To conduct a more comprehensive comparison, we considered patients with multiple-site metastases to have liver metastases, though liver metastases in these patients accounting for over 90%, it introduced some uncertainty. However, when comparing results from patients with liver-limited metastases and patients with multiple-site metastases, the conclusions were consistent. Therefore, we believe the impact of this assumption is limited. Lastly, the relative efficacy between many treatment regimens was obtained through indirect comparison, and more evidence from head-to-head RCTs is needed to validate our findings. Although the combination of anti-VEGF drugs with systemic therapy has been studied for its improvement of survival rates, our research further validates this finding. The value of this study lies in the integrated analysis of existing data, providing valuable insights for future research and clinical decision-making.

## 5 Conclusions

For CRLM patients who have failed at least one line of previous systemic therapy, multi-targeted therapy and targeted therapy plus chemotherapy are the best mechanisms. TAS-102 plus bevacizumab is superior in OS, and the combination of anti-VEGF drugs like bevacizumab and aflibercept with standard chemotherapy is the preferred option.

## Data availability statement

The original contributions presented in the study are included in the article/**Supplementary Material**. Further inquiries can be directed to the corresponding authors.

## Author contributions

YJ: Conceptualization, Data curation, Formal analysis, Investigation, Methodology, Software, Writing – original draft, Writing – review & editing. MZ: Data curation, Formal analysis, Investigation, Methodology, Writing – original draft, Writing – review & editing. WT: Funding acquisition, Resources, Software, Supervision, Validation, Writing – review & editing. XZ: Project administration, Resources, Software, Supervision, Validation, Writing – review & editing.
